# The effectiveness of an online intervention in preventing excessive gestational weight gain: the e-moms roc randomized controlled trial

**DOI:** 10.1186/s12884-018-1767-4

**Published:** 2018-05-09

**Authors:** Christine M. Olson, Susan W. Groth, Meredith L. Graham, Jennifer E. Reschke, Myla S. Strawderman, Isabel Diana Fernandez

**Affiliations:** 1 Division of Nutritional Sciences, 406 Savage Hall, Cornell University, Ithaca, NY 14853 USA; 2 School of Nursing, University of Rochester, Box SON, 601 Elmwood Ave., Rochester, NY 14642 USA; 3 Division of Nutritional Sciences, 352 MVR Hall, Cornell University, Ithaca, NY 14853 USA; 4 Department of Public Health Sciences, University of Rochester School of Medicine and Dentistry, 265 Crittenden Blvd, CU420644, Rochester, NY 14642 USA

**Keywords:** Gestational weight gain, Behavior change, Online intervention, Pregnancy, Randomized controlled trial

## Abstract

**Background:**

Excessive gestational weight gain (GWG) is common and contributes to the development of obesity in women and their offspring. Electronic or e-health interventions have the potential to reach large groups of women and prevent excessive GWG, but their effectiveness has not been demonstrated. The purpose of this study was to evaluate, in a real-world setting, the effectiveness of a self-directed, integrated online and mobile phone behavioral intervention in preventing excessive GWG.

**Methods:**

This effectiveness trial was a double-blind, three-arm trial with a parallel group design. Two arms received the same e-health intervention during pregnancy with the third arm serving as the placebo control. The intervention was based on a previously efficacious non-digital intervention that was adapted to electronic format. It included three behavior change tools: a weight gain tracker, and separate diet and physical activity goal-setting and self-monitoring tools. Both treatment conditions received access to informational tools, event reminders, and a blogging feature. Healthy pregnant women age 18-35 years with body mass indexes (BMI) ≥18.5 and < 35, at ≤20 weeks gestation, and an e-mail address were eligible. The proportion of women with excessive total GWG, as defined by the Institute of Medicine (IOM), was the primary outcome. 1689 randomized women were analyzed in the intent-to-treat (ITT) analysis. The study was designed to have 87% power to detect a 10 percentage point reduction from a control rate of 55% with a sample of 1641 (*p* = 0.0167, two-sided).

**Results:**

In the ITT sample, 48.1% (SD = 2.0%) gained excessively in the intervention group as did 46.2% (SD = 2.4%) in the placebo control group. These proportions were not significantly different (RR 1.09; 95% CI 0.98, 1.20, *p* = 0.12). The results were not altered in several sensitivity analyses.

**Conclusion:**

The addition of three behavior change tools to an informational placebo control did not result in a difference in the proportion of women with excessive total GWG compared to the placebo control in this effectiveness trial of an online, self-directed intervention. The similarity of intervention and control treatments and low usage of the behavior change tools in the intervention group are possible explanations.

**Trial registration:**

NCT01331564, ClinicalTrials.gov.

**Electronic supplementary material:**

The online version of this article (10.1186/s12884-018-1767-4) contains supplementary material, which is available to authorized users.

## Background

Excessive gestational weight gain (GWG) is a risk factor for postpartum weight retention that contributes to long-term weight gain in women [1–3]. It is also related to increased risk of obesity in offspring [2, 4]. In the US in 2015, about 39% of normal weight, 61% of overweight, and 55% of obese women, gained more than the recommended amount of weight during pregnancy [5]. Obesity experts have called for obesity prevention to begin during pregnancy and early infancy [6].

A recent Cochrane review found diet and/or exercise interventions during pregnancy reduced the risk of excessive GWG by 20% [7]. The estimate was robust and supported by high-quality evidence. Electronic communication technologies have the potential to reach large numbers of women during pregnancy with behavioral interventions to prevent excessive weight gain. However, there is currently a paucity of data on their effectiveness for this purpose [8, 9]. There is a growing body of evidence that electronic communication interventions, often called e- and m-health interventions, are efficacious in several different domains of health behavior change including weight loss and weight gain prevention among non-pregnant individuals [10, 11].

The aim of this study was to evaluate the effectiveness of a self-directed, integrated mobile phone and online behavior change intervention in preventing excessive GWG in a real-world setting.

## Methods

### Overview and design

**Fig. 1:**
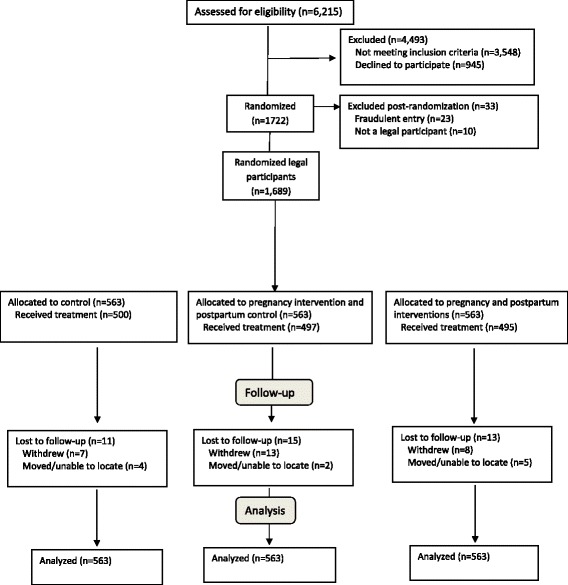
Participant flow diagram. ^a^ Fraudulent entries were multiple entries from women who were seeking to enter the study multiple times for monetary purposes. ^b^ Illegal participants were females who were too young to give informed consent or women whose signed consent forms were missing

The overall goal of the project of which this study is a part was to expand the understanding of how to slow the accumulation of weight in childbearing women. This paper reports on the pregnancy portion of a longer study that followed women until 12 months postpartum in order to evaluate the effectiveness of pregnancy intervention and combined pregnancy and postpartum intervention on weight retention at 12 months postpartum. Thus during pregnancy, two arms received the same intervention (one of these arms becomes a control postpartum) and the third arm was the placebo control (Fig. 1). The primary hypothesis for the pregnancy portion of the study was that the intervention would lead to a 10 percentage point reduction in the proportion of women with excessive total GWG in the intervention arms (45%) compared to the placebo control arm (55%).

This trial was a double-blind, three-arm randomized trial with a parallel group design with the individual as the unit of randomization and analysis [12]. Pregnant women were screened by research staff in prenatal clinics, private obstetric practices, ultra-sound offices, and over the phone and online in a large Northeastern US city from May 2011 through July 2012. Inclusion criteria were age 18-35 years and gestational age ≤ 20 weeks at time of enrollment. Exclusion criteria included body mass index (BMI) < 18.5 and ≥ 35 kg/m^2^, multiple gestation, weight-affecting medical or psychiatric conditions, and no e-mail address [12]. The age limits were set by the Early Adult Reduction of Weight through LifestYle interventions (EARLY) consortium of weight management studies of which this trial was a part [13]. Eligible women provided written informed consent and signed a form for release of their medical records. Upon consent, participants were electronically randomized via computer to 2 intervention arms and 1 control arm within 4 income (with low income defined by Medicaid eligibility) and BMI (normal BMI and overweight plus obese class 1 BMI) strata in blocks of six: strata 1 - normal BMI low income; strata 2 - normal BMI high income; strata 3 - overweight plus obese class 1 low income; and strata 4 - overweight plus obese class 1 high income. The study protocol was approved by the University of Rochester Research Subject Review Board and the Cornell University Institutional Review Board.

### Intervention

**Fig. 2:**
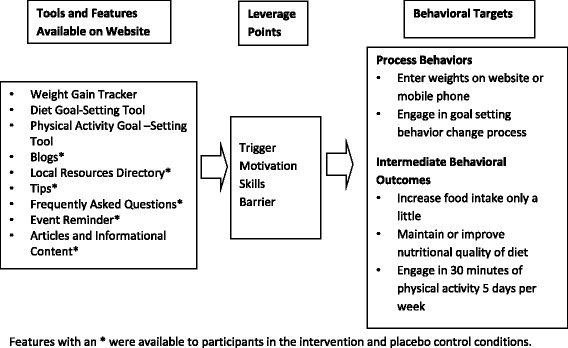
Website features, leverage points and behavioral targets for intervention

**Fig. 3:**
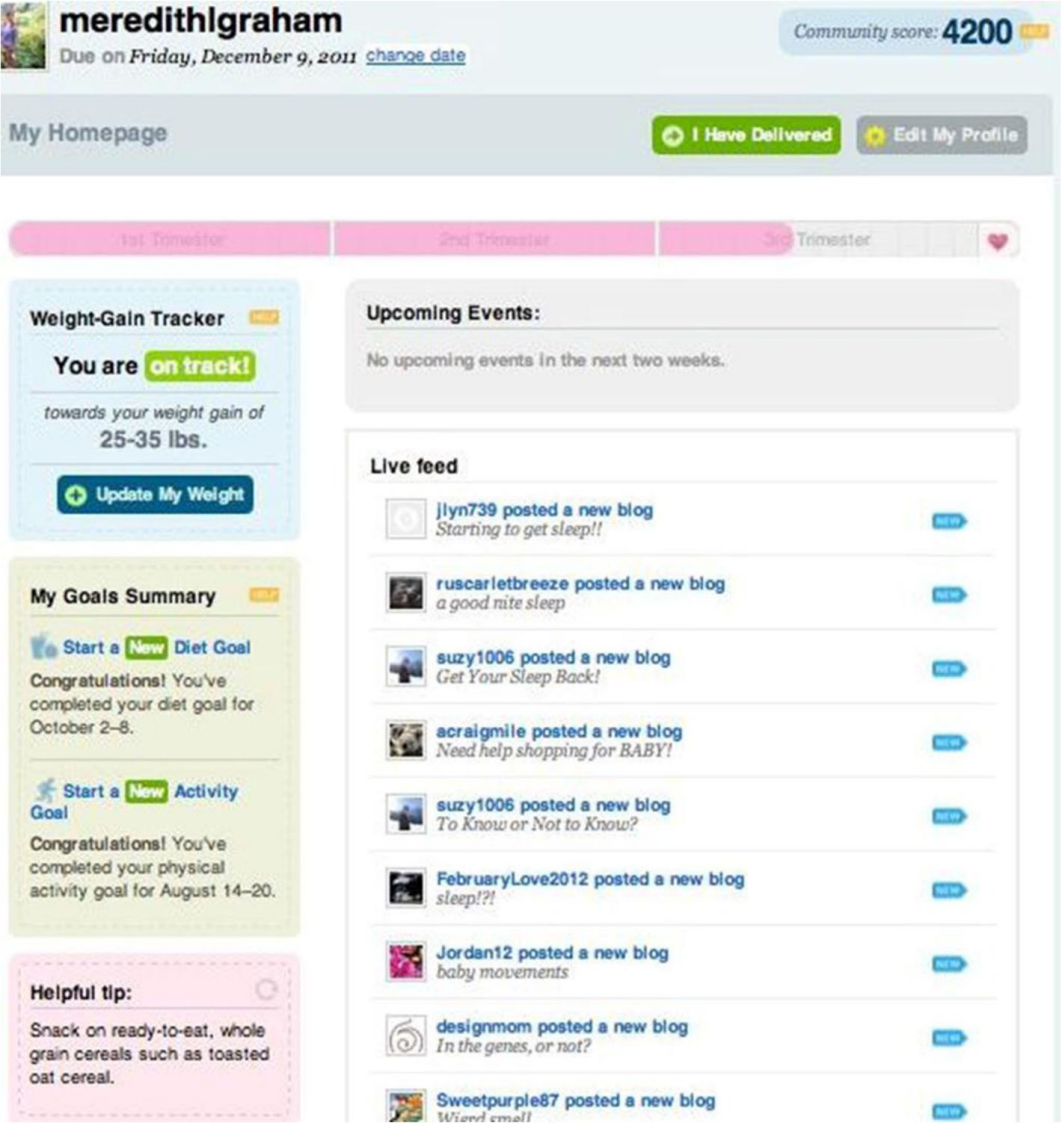
Dashboard for women on intervention arm

Participants assigned to the intervention arms received access to the intervention website and those assigned to the placebo control condition received access to the control website, both of which were password protected. A more detailed description of the theory, development and implementation of the pregnancy intervention is available [14, 15]. Briefly, the self-directed, integrated online and mobile phone behavioral intervention was designed using the Integrative Model of Behavior Prediction [16] and the Behavior Model for Persuasive Design [17] in addition to formative research with the target population [14]. It was based on a non-electronic pregnancy lifestyle intervention that was demonstrated to be efficacious in low-income women [18]. Women in the intervention arms received access to three behavior change tools including a weight gain tracker, a diet and a physical activity goal-setting and self-monitoring tool, as well as, health information including tips, articles, frequently asked questions; a description of pregnancy and parenting-related resources available in the local community; a blogging tool; and an event and appointment reminder. Fig. 2 shows these tools, their behavioral targets and leverage points from the theoretical model [17]. Fig. 3 shows the dashboard available to participants in the intervention group. The placebo control arm received access to all the features above except the weight gain tracker and the diet and physical activity goal-setting and self-monitoring tools since the latter were hypothesized to be the active ingredients of the intervention. Reminders and informational content, that differed by arm, were distributed weekly via e-mail messages to all participants. In summary, two different suites of tools were made available to trial participants on a password protected study website and mobile phone platform. Women were reminded weekly to login, and they decided what, when, and how much they would use the tools made available to them. All women in the trial received standard prenatal care from their self-selected health care provider.

### Measures and data collection

Data were collected at screening by research staff, with two online surveys completed by participants during pregnancy (one before 28 weeks gestation and one from 32 weeks until delivery), and through an audit of the women’s prenatal, labor and delivery, and 6 week postpartum medical records by research staff. Socio-demographic, behavioral, psychosocial and adverse event data were collected through online surveys [12]. Health and weight data were abstracted from the medical record [12]. Participants received financial incentives for enrolling in the study and for each of the online questionnaires they completed. They could earn up to $45 for completing all data collection activities during pregnancy. They were not paid for engaging with intervention or control treatments. An online satisfaction survey using EARLY questions and for which no financial incentive was provided was made available to participants to complete at 6 weeks postpartum [13].

The pre-specified primary outcome for evaluating the effectiveness of the intervention was the proportion of women with total GWG above the upper limit of the range for total GWG defined by the Institute of Medicine (IOM) for each BMI group [1]. Total GWG was calculated as the difference between the first weight at < 14 weeks gestation and the last weight at ≥37 weeks in pregnancy. The binary outcome, the proportion of women with excessive total GWG, was determined by comparing the difference for each woman to the IOM upper limit for GWG range for each BMI group: normal BMI - > 16 kg; overweight BMI - > 11.5 kg; and obese class 1 BMI - > 9 kg [1]. Excessive average weekly GWG in the last half of pregnancy and total GWG in kg were pre-specified secondary outcomes. Average weekly GWG was calculated as the difference between the last weight at ≥37 weeks of gestation and the weight nearest to 20 weeks gestation (+/− 2 weeks) divided by the number of weeks between the two weights. This value was defined as excessive if it exceeded the upper limit for weekly weight gain for each BMI group as specified by the IOM [1]. Adherence to the treatment protocol was defined as logging into the treatment arm specific project website at least once in each 45 day interval during pregnancy. This time interval was based on the schedule of prenatal care visits which are on average every 30 days during pregnancy. Formative research showed that a large proportion of women did not have scales in their homes and thus in order to use the weight gain tracker they needed to use the weights measured at their prenatal care visits [14]. This level of adherence was considered as providing a minimal possibly effective dose of exposure to treatment.

### Sample size

The target sample size of 1641 (547/arm) was determined by the weight retention endpoint at 12 months postpartum, the ending time point for the overall study [12]. Assuming 15% attrition during the pregnancy period, yielding 465 controls and 930 intervention subjects, we estimated 87% statistical power to detect a reduction of 10 percentage points from the overall expected 55% of women with excessive total GWG in the control group. The expected rate was calculated from data made available through the Finger Lakes Perinatal Data System which aggregates birth certificate information from hospitals in the region. (See Additional file 1: Table S1.) The study was not powered to examine intervention effects within strata. A Bonferroni-corrected significance level of 1.67% (2-sided), reflecting the three primary comparisons in the entire study, was applied to the primary analysis of excessive total GWG. Otherwise, no multiple comparison procedures were applied and a significance level of 5% was used.

### Statistical analysis

Missing data were handled using multiple imputation to address issues of bias which may result from analyzing only complete cases [19]. Sufficient weight information for the calculation of the primary outcome required having a measured weight at both < 14 weeks and ≥ 37 weeks of gestation. If weight information was insufficient, the first, 20 week, and/or last weights were imputed using Statistical Analysis System (SAS) Proc MI. A previous evaluation of the non-electronic version of the intervention indicated that both income and BMI affected GWG outcomes, leading to the stratified randomization design for the present study [18]. Therefore, to allow for a potential interaction between income, BMI, and intervention within the imputed data, 60 imputed data sets were created within each income and BMI group and used in the analysis.

The overall proportion of women with excessive GWG on each arm was estimated by pooling across the imputed data sets, accounting for the variability between imputations, as described by Ratitch, Lipkovich and O’Kelly [20]. Relative risk (RR) estimates for the binary outcomes (proportion of women with excessive total and weekly GWG) were obtained from log-binomial regression models. In two of the sensitivity analyses, some of the log-binomial models did not converge in a few of the 60 imputed data sets. The COPY method was then applied to address the computational issues [21] and was successful in addressing convergence issues in all cases. Mean differences in total GWG (kg) were similarly estimated from least-squares regression models. All multivariable model estimates for the overall effect of intervention versus control arms were adjusted for strata, early pregnancy BMI, gestational age at delivery (to adjust for length of gestation and thus the amount of time over which weight could be gained), and two variables to additionally adjust for the timing of weight measurements: the number of weeks between the first and last pregnancy weight, and the number of weeks between the last pregnancy weight and delivery. SAS Proc MIANALYZE was then used to combine the model results from within each imputed data set in the standard way according to Rubin’s Rules. The interaction between treatment arm and the four category strata variable was assessed as a secondary analysis. Several sensitivity analyses were conducted with various subsamples of study participants.

Descriptive information is presented on engagement within treatment arm. Satisfaction with study participation was compared by treatment arm using chi-square analysis. To describe the safety of the intervention, 10 infant and maternal health outcomes and complications were compared by arm within each of the four strata using Fisher’s Exact Test. Birthweight was compared using the T-Test.

## Results

### Description of sample

Of the 6215 women screened, 945 (15.2%) declined participation and 3548 (57.1%) were not eligible (Fig. 1). Of the 1722 (27.7%) women randomized, 23 were fraudulent meaning they were not individual women. They were multiple entries from women who eluded checkpoints and entered the study multiple times for monetary purposes. In addition, 10 women misreported their ages and were too young to give informed consent or their signed consent forms were missing. A sample of 1689 pregnant women was included in the intention-to-treat (ITT) analysis (Fig. 1).

Participants’ socio-demographic characteristics were similar between randomized arms (Table 1). Overall, the sample was about 43% low income which means they qualified to received Medicaid (government subsidized prenatal care) during pregnancy and had household incomes < 185% of the Federal poverty threshold. In each treatment group, 53.4% of women had a BMI in the normal range and 46.6% were overweight or class 1 obese. The sample was about 64% non-Hispanic white with African Americans making up 21.1% of the control group and 23.5% of the intervention group. The sample recruited into the study had a higher rate of overweight and obese low income women compared to the population who met study eligibility criteria and delivered infants at the four participating hospitals in the study area (22.3% vs. 15.3%) and had a lower rate of normal BMI high income women compared to the population (32.3% vs. 40.6%). Otherwise, the sample was similar to the population (Additional file 1: Table S1).

**Table 1:** Baseline characteristics of sample by treatment arm (*n* = 1689)

Characteristic^a^	Placebo control *n* = 563	Intervention *n* = 1126
BMI Income Strata	*n* (%)	n (%)
Normal Low Income	115 (20.4)	242 (21.5)
Normal Not-Low Income	186 (33.0)	359 (31.9)
Overweight + Obese Low Income	125 (22.2)	252 (22.4)
Overweight + Obese Not-Low Income	137 (24.3)	273 (24.3)
Initial BMI (kg/m^2^)		
Median (25th, 75th percentile)	24.7 (21.9, 28.3)	24.7 (22.0, 28.6)
Age at entry		
18 to 24.99 y	167 (29.7)	358 (31.8)
25 to 29.99 y	205 (36.4)	366 (32.5)
30 to 34.99 y	191 (33.9)	402 (35.7)
Race and ethnicity		
Non-Hispanic white	340 (64.8)	667 (63.8)
Non-Hispanic African American	111 (21.1)	246 (23.5)
Hispanic/Latina	27 (5.1)	73 (7.0)
Other	47 (9.0)	60 (5.7)
Missing (*n*)	38	80
Parity		
Nulliparous	264 (47.1)	511(45.4)
Primiparous	172 (30.7)	378 (33.6)
Multiparous	125 (22.3)	236 (21.0)
Missing (*n*)	2	1
Education		
High school or less	91 (20.2)	200 (22.5)
Some college	133 (29.6)	258 (29.0)
College graduate	113 (25.1)	213 (23.9)
Graduate or professional degree	113 (25.1)	219 (24.6)
Missing (*n*)	113	236
Smoked in pregnancy		
Yes	66 (12.6)	114 (11.0)
No	457 (87.4)	923 (89.0)
Missing (*n*)	40	89
Home internet use		
Most days/week	335 (71.7)	665 (72.0)
A few times or less	132 (28.3)	259 (28.0)
Missing (*n*)	96	202

^a^Table entries are shown as frequency and percent of known values, n (%), unless specified differently. Subjects with missing values are shown in the missing row for each characteristic

### Engagement with treatment

Participants engaged with their treatment arm specific website beginning at a median of 12 weeks of gestation and 34.6% of the placebo control and 46.1% of the intervention arms logged into the website at least once in each 45-day interval of participation and thus were defined as adherent to treatment (Table 2). Control and intervention women had access to the study website a median of 199 and 196 days, respectively, and they logged in a median of 3.2% and 5.6% of those days. The median number of logins and web page views for the placebo control and intervention women were 6 and 10 for logins and 15 and 24 for page views, respectively, with 6 of the page views for behavior change tools in the intervention group.

**Table 2:** Engagement with treatment assignment (*n* = 1689)

Indicator of engagement^a^	Placebo control *n* = 563	Intervention *n* = 1126
Logged into study web site at least once, *n* (%)	473 (84.0)	946 (84.0)
Week of pregnancy of first login	11.8 (8.9, 15.4)	12.0 (8.6, 16.0)
Logged-in each 45 days of participation (adherent), *n* (%)	195 (34.6)	519 (46.1)
Number of days with access to website	199 (166, 220)	196 (161, 220)
Percent of access days with a login	3.2 (0.9, 6.7)	5.6 (0.2, 11.7)
Number of logins for treatment^b^	6 (2, 14)	10 (2, 24)
Number of web page views	15 (2, 48)	24 (3,62)
Number of web page views of control content	15 (2, 48)	17 (3, 48)
Number of web page views of behavior change tool content	0 (0, 0)	6 (0, 14)

^a^Table entries are median (25th percentile, 75th percentile) unless otherwise noted

^b^This excludes logins for questionnaire completion and other study administrative tasks

Additional file 2 Table S2 displays the exposure to and engagement with the information and social content (the placebo content) by treatment arm. The two arms were sent an equal number of weekly e-mail messages, at a median of 28*.* Overall, the two arms were very similar in their exposure to the placebo content.

Overall, 1169 women (69.2%) submitted a satisfaction survey with no difference in the completion rate by arm. Of these women, 930 (80%) completed the two satisfaction questions reported here. On the 0 to 10 point scale, the majority of participants (53.6%) in both arms scored a 9 or 10 on the item related to recommending the website to other pregnant women (Additional file 3: Table S3). A significantly greater proportion of women in the intervention group scored 9 or 10 on the item asking about enjoying participation in the trial compared to the placebo control group (45.5% vs 37.6%; *p* = 0.02).

### Gestational weight gain (GWG) outcomes

Forty-eight and one-tenth percent of women in the intervention arm gained excessively as did 46.2% of women in the placebo control arm (Table 3). In the adjusted analysis, no differences between treatment arms were found in the ITT sample for the primary and secondary outcomes: proportion with excessive total GWG, proportion with excessive weekly GWG, and total GWG in kg (RR 1.09; 95% CI 0.98, 1.20; *p* = 0.12; RR 1.00; 95% CI 0.94, 1.07; *p* = 0.90; mean difference 0.10 kg; 95% CI -0.58, 0.77; *p* = 0.78), respectively. The interaction of treatment with the four-category strata variable (strata 1 - normal BMI low income; strata 2 - normal BMI high income; strata 3 - overweight plus obese class 1 low income; and strata 4 - overweight plus obese class 1 high income) was not significant for any outcome (*p* = 0.19, *p* = 0.22, and *p* = 0.16, respectively). The sensitivity analyses are shown in Table 4 for both excessive total GWG and excessive weekly GWG. Since the results between the two outcomes do not differ, only those for the primary outcome, excessive total GWG, are described in the text. The eligible and participating (treated) sample (*n* = 1335) showed results that were similar to those in the ITT sample: RR 1.10; 95% CI 0.98, 1.22; *p* = 0.09. Additional analyses with the complete case sample (*n* = 1337) for whom no weights were imputed and the per-protocol sample (*n* = 714) that only included women who were defined as adherent by logging in at least once in each 45 day interval of participation, also showed no significant difference between treatment arms. A sensitivity analysis that used self-reported pre-pregnancy weight rather than first measured weight at < 14 weeks gestation for the calculation of gestational weight gain showed no intervention effect, nor did an analysis that included only those who had not gained excessively at the time they started treatment (Table 4).

**Table 3:** Primary and secondary gestational weight gain (GWG) outcomes in the ITT sample

	Intervention^a^ *n* = 1126	Placebo control^a^ *n* = 563	Adjusted estimate^b^ (95% CI)	*P*-value
Primary outcome - % exceeding the upper limit of guidelines for total GWG				
Intervention effect	48.1% (2.0%)	46.2% (2.4%)	1.09 (0.98, 1.20)	0.12
Intervention x Strata interaction (3df)				0.19
Secondary outcome - % exceeding the upper limit of weekly GWG rate (kg/week)				
Intervention effect	66.4% (2.0%)	67.9% (2.3%)	1.00 (0.94, 1.07)	0.90
Intervention x Strata interaction (3df)				0.22
Secondary outcome analysis- total GWG (kg)				
Intervention effect	13.73 (0.46)	13.73 (0.45)	0.10 (−0.58, 0.77)	0.78
Intervention x Strata interaction (3df)				0.16

^a^Results are pooled across imputed data sets and are unadjusted for other factors (*n* = 1689)

^b^Relative Risk (RR) estimates of excessive total and weekly GWG from log-binomial model for intervention vs placebo adjusted for strata, gestational age at delivery, continuous BMI, and two timing of weight measurement variables. For total GWG, the mean difference (kg) between intervention and placebo from least squares regression model was adjusted for strata, gestational age at delivery, continuous BMI, and two timing of weight measurement variables. The COPY method was used if any of the 60 log-binomial models did not converge [20]

**Table 4:** Sensitivity analyses for excessive total gestational weight gain (GWG) and excessive weekly GWG

Sensitivity Sample	Outcome	Intervention^a^	Placebo control^a^	Adjusted estimate^b^ (95% CI)	*P*-value
Eligible and participating sample^c^	Excessive total GWG	47.6% (1.7%)	45.9% (2.4%)	1.10 (0.98, 1.22)	0.09
	Excessive weekly GWG	66.8% (1.6%)	68.9% (2.3%)	0.99 (0.93, 1.07)	0.85
Complete case sample^d^	Excessive total GWG	47.0%	45.1%	1.11 (1.00, 1.24)	0.06
	Excessive weekly GWG	67.0%	68.4%	1.01 (0.94, 1.08)	0.85
Per-protocol sample^e^	Excessive total GWG	45.5% (2.3%)	41.6% (3.6%)	1.14 (0.96, 1.37)	0.14
	Excessive weekly GWG	64.2% (2.2%)	62.2% (3.7%)	1.07 (0.95, 1.20)	0.28
As treated sample^f^	Excessive total GWG	47.4% (1.8%)	46.6% (2.2%)	1.08 (0.97, 1.2)	0.15
	Excessive weekly GWG	66.6% (1.7%)	68.8% (2.0%)	1.00 (0.93, 1.07)	0.89
Self-reported pre-pregnancy weight^g^	Excessive total GWG	53.9% (1.9%)	53.1% (2.4%)	1.05 (0.96, 1.15)	0.26
	Excessive weekly GWG	Not applicable	Not applicable	Not applicable	Not applicable
Not excessive GWG at start of treatment^h^	Excessive total GWG	42.8% (2.0%)	39.3% (2.7%)	1.12 (0.96, 1.30)	0.15
	Excessive weekly GWG	65.6% (2.0%)	66.3% (2.6%)	1.02 (0.93, 1.11)	0.68

^a^Results are pooled across imputed data sets (except for the complete case sample) and are not adjusted for other factors, [Mean % (SD)]

^b^Relative Risk (RR) estimates of excessive GWG from log-binomial model for intervention vs placebo adjusted for strata, gestational age at delivery, continuous BMI, and two timing of weight measurements variables. The COPY method was used if any of the 60 log-binomial models did not converge

^c^Eligible and participating sample: *N* = 1335; Intervention arm (*n* = 898), Placebo control arm (*n* = 437)

^d^Complete case sample for excessive total GWG: *N* = 1337; Intervention arm (*n* = 891), Placebo control arm (*n* = 446). Complete case sample for excessive weekly GWG rate: *N* = 1364; Intervention arm (*n* = 912), Placebo control arm (*n* = 452)

^e^Per protocol sample comprised of subjects who logged into website within each 45 day interval of participation: *N* = 714; Intervention arm (*n* = 519), Placebo control arm (*n* = 195)

^f^The as treated sample moves all those in the intervention arm who did not login or when they logged in, did not view the behavior change tools to the placebo control condition: *N* = 1335; Intervention arm (*n* = 788), Placebo control arm (*n* = 547)

^g^ITT sample with excessive GWG determined using self-reported pre-pregnancy weight rather than first measured weight ≤ 14 weeks gestation

^h^ITT sample not excessive GWG by first login to website (nearest weight within 6 weeks): *N* = 1055; Intervention arm (*n* = 709), Placebo control arm (*n* = 346)

### Safety and clinical outcomes

Two of the 40 maternal and neonatal outcomes and complications compared by treatment arm within strata differed in the eligible and participating sample: the proportion of cesarean deliveries that were repeat cesarean deliveries was significantly greater in the intervention than the control arm (92.3% vs. 66.7%; *p* ≤ 0.05) in strata 4 (overweight plus obese class 1 high income) and macrosomia (birthweight > 4000 g) was significantly less common in the intervention than control arm (6.8% vs. 14.0%; p ≤ 0.05) in strata 2 (normal BMI high income) (Additional file 4: Table S4). Thus, there is little evidence indicating the intervention harmed women or infants.

## Discussion

A socio-economically and racially diverse sample of women that was similar in terms of income and BMI to the population from which it was recruited entered into the study. The integrated online and mobile phone intervention that included 3 behavior change tools had no significant effect beyond the informational placebo control on any of the primary or secondary GWG outcomes. The result was robust to several sensitivity analyses.

In effectiveness trials with self-directed, online interventions focused on preventing excessive GWG, the level of engagement required to achieve a successful outcome is not known. In this trial, the level of engagement with the treatment arm specific website, also called adherence, was determined by research participants. In the intervention arm women, adherence at a level considered to be a minimum possibly effective dose was 46.1% (Table 2), despite the weekly e-mail reminders to login and view new content on the website that was also listed in the message.

Currently five relatively small pilot or feasibility efficacy trials in the literature used mobile and web-based communication technologies to address excessive GWG [22–26]. Among those with positive results, engagement with behavior change interventions was higher than for this study, ranging from 61 to 86%. These findings suggest that the low prevalence of adherence in the intervention arm may have contributed to the null findings of this effectiveness trial with its self-directed, online intervention.

While the placebo control and intervention participants had a similar number of web page views of informational content (the majority of page views), the intervention arm members had only 6 page views of the behavioral change tools. In addition, 135 of the 1126 women in the intervention group (12%) only viewed placebo control content, essentially self-assigning themselves to the control group. The weight gain tracker was the most widely used behavior change tool with 70% of intervention arm women using the tool at least once [14] and 27.3% using it consistently across pregnancy [27]. In an analysis of usage of intervention website features, using the weight gain tracker consistently across pregnancy was associated with a reduction in the proportion of women with excessive total and weekly GWG and mean total GWG among not-low income women [27, 28]. These results support the supposition that the low prevalence of consistent engagement with a key behavior change tool, the weight gain tracker, was not sufficient to produce a difference in weight outcomes between treatment arms and demonstrate the challenge of implementing the IOM guidelines in public health contexts.

Pregnancy has been described as a teachable moment when women are highly motivated to engage in healthy behaviors [29]. For motivated women in the placebo control arm, the information and blogging tools may have been sufficient for preventing excessive GWG. Another RCT with a health coaching intervention to prevent excessive GWG that had an education only control condition also saw no difference in weight gain outcomes between treatment groups [30]. However, no associations were found in this trial between the amount of use of the website and weight outcomes in the placebo control arm decreasing the likelihood of this explanation for the null findings [28].

In this study, personal contact with study staff was limited and similar across treatment arms. A recent review of mobile phone interventions for weight management has shown that the amount of personal contact was significantly (*p* = 0.05) related to differences in weight outcomes [31].

This study has several limitations related to the measurement of weight outcomes and the design of the intervention. This study used a weight < 14 weeks gestation from women’s medical records for calculating total GWG. These medical records came from 29 different practices and clinics likely leading to measurement error and inflating the variability in GWG. However, the sensitivity analyses showed that using self-reported pre-pregnancy weights for calculating GWG, as is done in most studies of this topic, did not alter trial outcomes, indicating that the lack of an intervention effect is not highly sensitive to measurement error.

The study relied on self-reported weight and height for calculating BMI for strata assignment and identifying the upper limit for GWG for the weight gain tracker. If women under-reported their pre-pregnancy weight, which was likely, that would lead to a lower BMI and potential misclassification by BMI group. The results would be that under-reporting women would have been told by the study website to gain too much weight during pregnancy. This would decrease the likelihood that the intervention would have a positive effect compared to no weight advice given to the placebo control condition. In future trials, women should be weighed at entry in early pregnancy by research staff in order to correctly identify the upper limit of GWG and avoid giving incorrect advice on appropriate GWG.

Another limitation of the study was that the intervention was self-directed. This was done to meet the very diverse needs of the population of women [32] and to mimic real-world conditions for this translational research trial. The goal was to provide an intervention that was similar to what women could find on their own in the online environment. This may have been an unwise decision. A recent review of the literature on e-behavioral nutrition interventions showed that structured, tailored interventions were more likely to be successful in achieving dietary change [33].

A major strength of this study that supports the importance of publishing this null findings paper is that it is the first large RCT to test a self-directed, integrated e- and m-health intervention to prevent excessive GWG in a real-world setting, much like what any woman could access on her own in an online environment. A related strength is that the trial included a socio-economically, racially, and ethnically diverse sample and used a theory-based suite of behavior change intervention features.

## Conclusion

This trial failed to show a positive effect of a self-directed, integrated online and mobile phone behavior change intervention on the proportion of the sample with excessive total GWG compared to an information only placebo control condition. The lack of effect was robust to several sensitivity analyses. There are likely multiple explanations for this result. Our view is that in the overall sample, the behavior change features in the intervention were used too little by too few women to produce a difference from the information only placebo control condition. The behavior change features, particularly the weight gain tracker, could possibly have had an effect if they had been more widely and consistently used across pregnancy. Finally, obtaining an accurate measured body weight very early in pregnancy is essential for giving the correct advice on GWG and for the calculation of the GWG outcome.

Several large trials are currently underway to evaluate the efficacy of various intervention approaches to preventing excessive GWG in overweight and obese women [34]. The results of these trials plus the results of this trial have the potential to enhance the understanding of how to utilize e-and m-health interventions in population-wide obesity prevention programs during pregnancy.

## Additional files

Additional file 1: **Table S1.** Description of population and intent-to-treat (ITT) sample by strata; text (PDF 184 kb)

Additional file 2: **Table S2.** Engagement with informational content by treatment arm; text (PDF 191 kb)

Additional file 3: **Table S3.** Proportion of participants with very high satisfaction scores (9 or 10) by treatment arm; text (PDF 192 kb)

Additional file 4: **Table S4.** Infant and maternal health outcomes and complications; text (PDF 268 kb)

## Abbreviations

BMI: Body mass index**,**
CI: Confidence interval
EARLY: Early Adult Reduction of Weight through LifestYle
GWG: Gestational weight gain
IOM: Institute of Medicine
ITT: Intention to treat
kg: kilograms
m: meters
RR: Relative risk
SAS: Statistical Analysis System
SD: Standard deviation
US: United States
y: years
