# Supplementary material for: The effectiveness of an online intervention in preventing excessive gestational weight gain: the e-moms roc randomized controlled trial
Source: BMC Pregnancy Childbirth. 2018 May 9;18:148. doi: 10.1186/s12884-018-1767-4 (PMC5944067; doi:10.1186/s12884-018-1767-4)
Supplement: Supplementary file 1 — Table S1. Description of population and intent-to-treat (ITT) sample by strata; text (PDF 184 kb) [file 12884_2018_1767_MOESM1_ESM.pdf]

Supplemental Table 1 Description of population and intent-to-treat (ITT) sample by strata

BMI Category	Income	Population from FLPDS[a]	e-Moms-Roc ITT Sample
**Percent of Population and Sample**			
Normal (18.5-24.9)	Low	903 (19.6%)	357 (21.1%)
	High	1873 (40.6%)	545 (32.3%)
Overweight + Obese (25.0-35.0)	Low	705 (15.3%)	376 (22.3%)
	High	1131 (24.5%)	410 (24.3%)
Total		4612 (100%)	1689 (100%)
**Percent with Excessive Gestational Weight Gain[b]**			
Normal	Low	419 (46.4%)	42/94 (44.7%)
	High	845 (45.0%)	62/151 (41.1%)
Overweight + Obese	Low	439 (62.3%)	58/91 (63.7%)
	High	838 (74.1%)	76/110 (69.1%)
Total		2541 (55.1%)	238/446 (53.4%)

[a] Women giving birth in 2007 from Finger Lakes Perinatal Data System (FLPDS) meeting following e-Moms eligibility criteria: Delivery at participating hospitals; age 18-35 years; gestational age at delivery 37 - 42 weeks; singleton birth; maternal pre-pregnancy BMI 18.5 - <35.0; and no weight-affecting health conditions.

[b] These proportions use self-reported pre-pregnancy weight for the calculation of gestational weight gain. The e-Moms Roc proportions are calculated for the complete case sample subsample (n = 446) in the control arm.
