# Supplementary material for: The effectiveness of an online intervention in preventing excessive gestational weight gain: the e-moms roc randomized controlled trial
Source: BMC Pregnancy Childbirth. 2018 May 9;18:148. doi: 10.1186/s12884-018-1767-4 (PMC5944067; doi:10.1186/s12884-018-1767-4)
Supplement: Supplementary file 2 — Table S2. Engagement with informational content by treatment arm; text (PDF 191 kb) [file 12884_2018_1767_MOESM2_ESM.pdf]

Supplemental Table 2 Engagement with informational content by treatment arm (n = 1689)

Indicator of Engagement	Placebo Control n = 563	Intervention n = 1,126
Number of weekly e-mail messages sent, Median (25th,75th)	28 (23, 31)	28 (23, 31)
Number of events set using calendar feature, n (%)		
no events	416 (73.9)	891 (79.1)
1-5 events	112 (19.9)	194 (17.2)
> 5 events	35 (6.2)	41 (3.6)
Article views, n (%)		
None	275 (48.9)	651 (57.8)
1- 5 (median) [a]	142 (25.2)	265 (23.5)
>5	146 (25.9)	210 (18.7)
Article votes, n (%)		
None	493 (87.6)	1018 (90.4)
1- 2 (median)	35 (6.2)	56 (5.0)
>2	35 (6.2)	52 (4.6)
Article comments, n (%)		
None	509 (90.4)	1061 (94.2)
1 (median)	28 (5.0)	39 (3.5)
>1	26 (4.6)	26 (2.3)
FAQ views, n (%)		
None	351 (62.3)	782 (69.5)
1- 4 (median)	105 (18.7)	190 (16.9)
>4	107 (19.0)	154 (13.7)

FAQ votes, n (%)		
None	523 (92.9)	1065 (94.6)
1- 2 (median)	23 (4.1)	35 (3.1)
>2	17 (3.0)	26 (2.3)
Resource views, n (%)		
None	318 (56.5)	729 (64.7)
1- 4 (median)	132 (23.5)	216 (19.2)
>4	113 (20.1)	181 (16.1)
Resource votes, n (%)		
None	506 (89.9)	1025 (91.0)
1- 2 (median)	31 (5.5)	61 (5.4)
>2	26 (4.6)	40 (3.6)
Resource comments, n (%)		
None	517 (91.8)	1057 (93.9)
1 (median)	28 (5.0)	41 (3.6)
>1	18 (3.2)	28 (2.5)
Blog entries, n (%)		
None	479 (85.1)	973 (86.4)
1- 2 (median)	59 (10.5)	99 (8.8)
>2	25 (4.4)	25 (4.8)
Blog views, n (%)		
None	262 (46.5)	551 (48.9)
1- 8 (median)	158 (28.1)	306 (27.2)
>8	143 (25.4)	269 (23.9)

Blog votes, n (%)		
None	495 (87.9)	1005 (89.3)
1- 2 (median)	36 (6.4)	63 (5.6)
>2	32 (5.7)	58 (5.2)
Blog comments, n (%)		
None	433 (76.9)	904 (80.3)
1- 3 (median)	68 (12.1)	118 (10.5)
>3	62 (11.0)	104 (9.2)

[a] Overall median number of actions was used to categorize counts, among those with any of the specific action.
