# Supplementary material for: The effectiveness of an online intervention in preventing excessive gestational weight gain: the e-moms roc randomized controlled trial
Source: BMC Pregnancy Childbirth. 2018 May 9;18:148. doi: 10.1186/s12884-018-1767-4 (PMC5944067; doi:10.1186/s12884-018-1767-4)
Supplement: Supplementary file 3 — Table S3. Proportion of participants with very high satisfaction scores (9 or 10) by treatment arm; text (PDF 192 kb) [file 12884_2018_1767_MOESM3_ESM.pdf]

Supplemental Table 3 Proportion of participants with very high satisfaction scores (9 or 10)[a] by treatment arm

	Placebo Control Arm (n=319)		Intervention Arm (n=611)		P-value[b]
	0-8	9-10	0-8	9-10	
**Total Sample (n=930)**					
Enjoyed participating	199 (62.38)	120 (37.62)	33 (54.50)	278 (45.50)	0.02
Would recommend	148 (46.39)	171 (53.61)	281 (46.14)	328 (53.68)	0.94
**Strata 1 – Normal BMI Low Income (n=151)**					
Enjoyed participating	35 (66.04)	18 (33.96)	38 (38.78)	60 (61.22)	<0.01
Would recommend	25 (47.17)	28 (52.83)	27 (27.84)	70 (72.16)	0.02
**Strata 2 – Normal BMI Not-Low Income (n=380)**					
Enjoyed participating	84 (64.12)	47 (35.88)	159 (63.86)	90 (36.14)	0.96
Would recommend	63 (47.73)	69 (52.27)	139 (55.38)	112 (44.62)	0.15
**Strata 3 – Overweight + Obese Class 1 Low Income (n=145)**					
Enjoyed participating	26 (52.00)	24 (48.00)	38 (40.00)	57 (60.00)	0.17
Would recommend	17 (35.42)	31 (64.58)	32 (33.68)	63 (66.32)	0.84
**Strata 4 – Overweight + Obese Class 1 Not-Low Income (n=254)**					
Enjoyed participating	54 (63.53)	31 (36.47)	98 (57.99)	71 (42.01)	0.40
Would recommend	43 (50.00)	43 (50.00)	83 (50.00)	83 (50.00)	>0.99

[a] Satisfaction was measured using questions from the EARLY consortium with a 0 to 10 Likert scale for responses [12].

[b] Chi-square test compared the proportion with satisfaction scores of 9 or 10 to 0 to 8 by randomized arm among those who completed the Pregnancy Satisfaction Survey.
