# Supplementary material for: The effectiveness of an online intervention in preventing excessive gestational weight gain: the e-moms roc randomized controlled trial
Source: BMC Pregnancy Childbirth. 2018 May 9;18:148. doi: 10.1186/s12884-018-1767-4 (PMC5944067; doi:10.1186/s12884-018-1767-4)
Supplement: Supplementary file 4 — Table S4. Infant and maternal health outcomes and complications; text (PDF 268 kb) [file 12884_2018_1767_MOESM4_ESM.pdf]

Supplemental Table 4 Infant and maternal health outcomes and complications

Outcome or complication	Strata 1 Normal BMI low income		Strata 2 Normal BMI high income		Strata 3 Overweight + obese BMI low income		Strata 4 Overweight + obese BMI high income	
	Control (n=86)	Intervention (n=179)	Control (n=161)	Intervention (n=319)	Control (n=82)	Intervention (n=172)	Control (n=108)	Intervention (n=228)
**Maternal birth outcomes**								
No birth information	2 (2.3)[a]	5 (2.8)	4 (2.5)	10 (3.1)	2 (2.4)	7 (4.1)	2 (1.9)	6 (2.6)
C-section[b]								
Total / evaluated	11/83 (13.3)	36/174 (20.7)	27/157 (17.2)	69/308 (22.4)	22/79 (27.9)	42/164 (25.6)	31/106 (29.3)	79/222 (35.6)
Primary/no prior C-section	6/76 (7.9)	21/158 (13.3)	19/148 (12.8)	46/281 (16.4)	14/69 (20.3)	21/137 (15.3)	23/94 (24.5)	42/183 (23.0)
Repeat/prior C-section	5/7 (71.4)	15/16 (93.8)	8/9 (88.9)	22/27 (81.5)	8/10 (80.0)	20/27 (74.1)	8/12 (66.7)	36/39 (92.3)[c]
Missing C-section type	0	0	0	1	0	1	0	1

**Maternal antenatal complications**	83	173	157	307	79	164	106	221
Preeclampsia and eclampsia	4 (4.8)	9 (5.2)	5 (3.2)	9 (2.9)	2 (2.5)	8 (4.9)	7 (6.6)	8 (3.6)
Maternal hypertension	3 (3.6)	5 (2.9)	5 (3.2)	11 (3.6)	4 (5.1)	9 (5.5)	8 (7.6)	10 (4.5)
Gestational diabetes	2 (2.4)	4 (2.3)	7 (4.5)	8 (2.6)	2 (2.5)	10 (6.1)	7 (6.6)	15 (6.8)
**Neonatal outcomes among deliveries ≥28 weeks**	84	174	157	309	80	165	106	222
Preterm delivery, <37 wks	4 (4.8)	7 (4.0)	9 (5.7)	8 (2.6)	6 (7.5)	9 (5.5)	7 (6.6)	17 (7.7)
Missing birthweight	1	0	0	2	3	1	1	1
Infant birth weight (g)	3,298±601	3,288±490	3,437±475	3,390±445	3,288±526	3,366±528	3,456±557	3,450±534
Birthweight < 2500 g	6 (7.2)	9 (5.2)	5 (3.2)	8 (2.6)	4 (5.2)	8 (4.9)	6 (5.7)	12 (5.4)
Birthweight >4000 g	7 (8.4)	11 (6.3)	22 (14.0)	21 (6.8)[c]	6 (7.8)	15 (9.2)	15 (14.2)	28 (12.6)

[a] Numbers in parentheses within body of table are %.

[b] C-section is Caesarian section. Each entry is shown with appropriate denominator for the type of C-section.

[c] Fisher's exact test for comparison of control and intervention arms within strata with 2-sided significance level ≤ 0.05. Results without superscript are not statistically significant.
